# Antiobesity Effects of Hydroethanolic Extract of* Jacaranda decurrens* Leaves

**DOI:** 10.1155/2016/4353604

**Published:** 2016-12-12

**Authors:** Kátia Avila Antunes, Débora da Silva Baldivia, Paola dos Santos da Rocha, Junior Cesar Casagrande, Eliana Janet Sanjinez Argandoña, Maria do Carmo Vieira, Cláudia Andrea Lima Cardoso, Edson Lucas dos Santos, Kely de Picoli Souza

**Affiliations:** ^1^School of Environmental and Biological Science, Federal University of Grande Dourados, Rodovia Dourados Itahum, Km 12, 79804-970 Dourados, MS, Brazil; ^2^Faculty of Food Engineering, Federal University of Grande Dourados, Rodovia Dourados Itahum, Km 12, 79804-970 Dourados, MS, Brazil; ^3^Agricultural Sciences, Federal University of Grande Dourados, Rodovia Dourados Itahum, Km 12, 79804-970 Dourados, MS, Brazil; ^4^Course of Chemistry, State University of Mato Grosso do Sul, Rodovia Dourados Itahum, Km 12, 79804-970 Dourados, MS, Brazil

## Abstract

Obesity is a worldwide epidemic that reduces life expectancy; therefore, the search for new alternative and effective treatments is ongoing. The aim of the present investigation was to identify the chemical compounds in the hydroethanolic extract of leaves of* Jacaranda decurrens* subsp.* symmetrifoliolata* and to evaluate their toxicity and antiobesity effects. High-performance liquid chromatography was used to identify the chemical constituents, and acute toxicity was evaluated in rats treated with doses of 2 and 5 g·kg^−1^ body mass. The antiobesity effect was determined in rats with hypercaloric diet-induced obesity. Our results revealed the presence of compounds, such as jacaric, ursolic, and oleic acids, as well as luteolin, quercetin, and kaempferol, in the extract. The acute toxicity tests revealed that rats treated with elevated doses of the extract showed no signs of toxicity. The extract induced reduction in total body mass and the white adipose tissue depots. The obese rats treated with the extract showed an increased fluid intake and feces excretion while their serum total cholesterol and triglyceride levels decreased compared to those in the controls, without any hematological changes. Taken together, the results showed that the constituents of* J. decurrens* extracts included phenolic compounds and exhibited antiobesity effects with no toxicity.

## 1. Introduction

Epidemiological studies carried out by the World Health Organization (2013) indicated that 2.8 million adults died worldwide as a result of being obese and obesity significantly decreases the average lifespan. A disruption in energy homeostasis, which is the balance between energy gain and expenditure, is the key trigger in the development of obesity and involves both genetic and environmental factors. High-energy consumption, especially of lipids, is one of the main contributors to the accumulation of adipose tissue in addition to a reduction in physical activity and consumption of thermogenic foods [1].

Obesity is characterized by the hypertrophy of white adipose tissue (WAT) and is closely related to cardiovascular and metabolic alterations [2]. The accumulation of WAT is accompanied by an increase in proinflammatory adipokines [3] and structural alterations resulting from the oxidative stress caused by obesity [4].

Different constituents of vegetable origin have been used as alternative therapies for the treatment and prevention of obesity, including phenolic compounds. These substances are described as suppressors of fatty acid synthesis and lipolysis stimulators [5] and are known to reduce body mass, white adipose tissue, cholesterol, and serum and hepatic triglycerides [6]. In addition, phenolic compounds are reported to have antioxidant effects [7]. Although medicinal plants are generally considered to have low toxicological risks, some of their numerous constituents may show dose-dependent toxicity [8] and therefore need to be investigated.

The subshrub* Jacaranda decurrens* subsp.* symmetrifoliolata *Farias & Proença (Bignoniaceae) is one of the plants popularly used in traditional medicine in Brazil. It is found in the State of Mato Grosso do Sul, Brazil, and its biological potential has not been thoroughly explored. Therefore, the objectives of this study were to identify the chemical constituents as well as evaluate the acute toxicity and antiobesity effects of the hydroethanolic leaf extract of* J. decurrens* (ExJds) in Wistar rats with diet-induced obesity.

## 2. Material and Methods

### 2.1. Ethics Statement

*J. decurrens* leaves were collected following the identification of the plant and authorization of the Sistema de Autorização e Informação em Biodiversidade (SISBIO) (permit number 39451-1). This study was conducted in accordance with the Ethical Principles for Animal Experimentation adopted by the National Council for the Control of Animal Experimentation (CONCEA) and was approved by the Ethics Committee for Animal Use (CEUA) of the Federal University of Grande Dourados, Brazil, under protocol number 022/2012.

### 2.2. Botanical Material and Extract Preparation

*J. decurrens* leaves were collected in Dourados, Mato Grosso do Sul, Brazil (S 22° 02′ 49.1′′ and W 055° 08′ 11.4′′), oven-dried with air circulation at a temperature of 45 ± 5°C, and then ground in a Willy-type knife mill. An exsiccated sample was deposited in the Herbarium of the Federal University of Grande Dourados, Mato Grosso do Sul, Brazil, with registration number 2322.

The extract was then prepared by macerating the plant material in an ethanol : water (80 : 20, v/v) mixture at room temperature for 7 days. Then, the extract was filtered, and the residue was further extracted twice by using the same process. After 21 days, the filtrate was concentrated in a rotary vacuum evaporator (Fisatom, São Paulo, SP, Brazil) and freeze-dried to obtain a calculated specific yield of 12%; the final freeze-dried hydroethanolic extract of* J. decurrens* (ExJds) was stored at 4°C with protection from light.

### 2.3. Isolation and Quantification of Compounds

The ExJds (18 g) was dissolved in 0.5 L of water and fractionated using XAD-2 (Supelco, Bellefonte, PA, USA) resin column chromatography (30 cm × 3 cm) and eluted with 0.5 L of water, followed by 0.45 L of methanol, and finally with 0.3 L of ethyl acetate. We collected 73 fractions of 6 mL each from the methanolic extract of the leaves. The fractions were combined based on their profile in thin layer chromatography (silica gel plates, ethyl acetate : n-propanol : water, 120 : 7 : 70, v/v/v, upper phase). Fractions 43–46 resulted in the isolation of the compound kaempferol-3-O-a-l-rhamnopyranoside (3 mg). Fractions 6–18 and 21–27 were purified using polyvinylpolypyrrolidone (Sigma-Aldrich, St. Louis, MO, USA) column chromatography (10 × 2 cm) and eluted with methanol, which led to the identification of kaempferol-3-O-a-l-rhamnopyranosyl-(1-6)-b-d-glucopyranoside and quercetin-3-O-a-l-rhamnopyranosyl-(1-6)-b-d-glucopyranoside (3 and 2 mg), respectively. A 0.83-g aliquot of the ethyl acetate fraction of the leaves was dissolved in 5 mL of methanol and fractionated using a Sephadex LH-20 (Amersham Pharmacia Biotech, Uppsala, Sweden) column chromatography system (80 cm × 2 cm) and eluted with methanol at a flow rate of 0.3 mL·min^−1^; we collected 49 fractions of 6 mL each. The fractions were combined based on their thin layer chromatography (silica gel plates, methanol : ethyl acetate, 80 : 20, v/v) profiles. The compounds kaempferol, luteolin, and quercetin (yield = 5, 4, and 7 mg, resp.) were isolated from fractions 7–10, 20–23, and 29–34, respectively. The combined mixture of fractions 38–46 was purified using a liquid chromatography- (LC-) 18 column (1 g, particle size, 55–105 mm, 6 mL, Supelco, Bellefonte, PA, USA). Briefly, a 1 mL sample was added to the column and subsequently eluted with methanol (3 × 1 mL), acetone (3 × 1 mL), and hexane (4 × 1 mL), in that order, leading to the identification of ursolic acid (4 mg) in the acetone fraction and oleic acid (4 mg) in the hexane fraction. All the compounds were identified and their structures were confirmed by comparing experimental data (infrared [9], nuclear magnetic resonance [10], and mass spectrometry [11] data) with those previously reported in the literature.

The chromatographic analysis was performed using spectroscopy-grade acetonitrile purchased from Merck (Darmstadt). The stock solutions of the standards were prepared from individual solutions dissolved in acetonitrile and were used as external standards. Ursolic and oleic acids, luteolin, quercetin, and kaempferol (98%) were purchased from Sigma-Aldrich (St. Louis, MO, USA), and quercetin-3-O-a-l-rhamnopyranosyl-(1-6)-b-d-glucopyranoside, kaempferol-3-O-a-l-rhamnopyranoside, and kaempferol-3-O-a-l-rhamnopyranoside-(1-6)-b-d-glucopyranoside were isolated from the plant material and purified in the laboratory by using high-performance liquid chromatography. Jacaric acid was donated to the laboratory.

The extract and standards were analyzed using an analytical LC system (Varian 210) equipped with a photodiode array detector monitored at a wavelength (*λ*) range of 200–800 nm. The LC column was a C-18 column (25 cm × 4.6 mm; gel particle size, 5 *μ*m; Luna, Phenomenex, Torrance, CA, USA). The flow rate and injection volume were set at 1.0 mL·min^−1^ and 20 *μ*L, respectively, for each analysis. All the chromatographic analyses were performed at 22°C while elution was carried out using acetic acid (0.3%, solvent A) and acetonitrile (solvent B). The conditions for gradient elution were 85–80% (A) over 20 min; 80–60% (A) over 20–45 min, and 60–15% (A) over 45–99 min and return to the initial condition over 99–105 min.

Content estimation of the standards in the extract was performed by external calibration. Aliquots (20 *μ*L) of the diluted standards (1–20 *μ*g·mL^−1^) were analyzed using LC by plotting the mean of the chromatogram areas against the concentration to construct a standard calibration curve. The equation parameters (slope and intercept) of the calibration curve were used to obtain the concentrations of the compounds in the extract.

### 2.4. Acute Toxicity

The Wistar rats (*n* = 9) used in this experiment weighed approximately 250 g and were fed the control diet. They were subsequently divided into the following three experimental groups (*n* = 3 each) and treated as follows: (I) control (water), (II) ExJds-2 (ExJds, 2 g·kg^−1^ body weight), and (III) ExJds-5 (ExJds, 5 g·kg^−1^ body weight). The rats were each treated with a single dose by oral gavage and their food consumption, water ingestion, body weight, piloerection, and mortality were monitored for 15 days. After euthanasia, the organs (the liver, lung, kidney, and heart) were collected, separately weighed, and macroscopically analyzed. In addition, blood samples were collected for the determination of aspartate aminotransferase (AST), alanine aminotransferase (ALT), and urea levels by using the COBAS equipment (Roche).

### 2.5. Diets

The hyperlipidic chow was composed of 42, 13, and 42% lipids, proteins, and carbohydrates, respectively, with an energy value of 592 ± 12 kcal·100 g^−1^, which was obtained by adding lard to the commercial rodent chow (Labina). Drinking water was supplemented with 10% fructose, and the hyperlipidic diet plus water with 10% fructose constituted the hypercaloric diet. The caloric value of the commercial rodent chow was 333 ± 1 kcal·100 g^−1^. No fructose was added to the drinking water of the Wistar rats fed with the commercial chow, and this combination was considered as the control diet.

### 2.6. Experimental Design and Procedures

The Wistar rats were prefed the control (*n* = 5) or hypercaloric (*n* = 15) diets for 6 months and attained body weights of 477 ± 14 and 516 ± 11 g, respectively, at the end of this period. Subsequently, the rats were divided into the control (control diet plus water, *n* = 5) and hypercaloric diet (*n* = 15) groups, which were further subdivided into three experimental groups (*n* = 5 each) that received water (OB); sibutramine, 2 mg·kg^−1^ body weight (OB-Sibu); and ExJds, 400 mg·kg^−1^ body weight (OB-ExJds) by gavage for 60 days. Throughout the experimental period, the changes in body weight, food consumption, and water intake were monitored.

### 2.7. Evaluation of Fecal Lipid Excretion

After treatment for 7 weeks, the Wistar rats were housed in individual metabolic cages for 24 h with food and water* ad libitum *according to their experimental groups. Their feces were collected and weighed, and the results are presented as g·100 g^−1^ per body weight. Then, the feces were dried in an incubator at 40 ± 5°C for 24 h and ground; then, the lipid content was analyzed using Soxhlet extraction [12].

### 2.8. Organ and Tissue Evaluations

After euthanasia, the WAT depots (retroperitoneal, epididymal, mesenteric, and inguinal subcutaneous) and skeletal muscles (soleus and extensor digitorum longus), as well as the heart, kidney, and liver of the rats were separately weighed. Blood was collected, and biochemical (glycemia, total cholesterol, and triglycerides) and hematological (hematocrit, hemoglobin, and leukocytes) parameters were evaluated using the COBAS equipment.

### 2.9. Statistical Analysis

The data were expressed as the mean ± standard error of the mean (SEM). A one-way analysis of variance (ANOVA) and the* post hoc* Student-Newman-Keuls or Student* t*-tests were used for the analysis and comparison of the results of the different experimental groups. The data were considered significant when *P* < 0.05.

## 3. Results

### 3.1. Compounds Isolated and Identified in the ExJds

The identification of the isolated compounds (kaempferol-3-O-a-l-rhamnopyranoside, kaempferol-3-O-a-l-rhamnopyranosyl-(1-6)-b-d-glucopyranoside, quercetin-3-O-a-l-rhamnopyranosyl-(1-6)-b-d-glucopyranoside, kaempferol, luteolin, quercetin, ursolic acid, and oleic acid) was achieved by analyzing the infrared, nuclear magnetic resonance, mass spectrometry, and experimental data. The structures of the compounds were subsequently confirmed by comparing the experimental data with those previously reported in the literature [9–11].

Analysis of the compounds using photodiode array detector scanning at a spectral range of 200–800 nm revealed that the developed elution method did not interfere with the LC retention time of the ExJds. The standards were easily identified and quantified based on their absorption spectra in the ultraviolet region and retention times. Furthermore, the standards found in the extract were unambiguously identified by performing coinjection experiments in which aliquots of the extracts and standards were mixed, diluted to a known volume, and then analyzed using LC.

The coefficients of determination (*r* ^2^) were 0.9994 for ursolic acid, oleic acid, luteolin, quercetin, and kaempferol and 0.992 for quercetin-3-O-a-l-rhamnopyranosyl-(1-6)-b-d-glucopyranoside, kaempferol-3-O-a-l-rhamnopyranoside, and kaempferol-3-O-a-l-rhamnopyranoside-(1-6)-b-d-glucopyranoside. The concentrations of the compounds were expressed as microgram of compound per gram of the extract and the values were as follows: ursolic acid, 184 *μ*g·g^−1^; oleic acid, 201 *μ*g·g^−1^; luteolin, 156 *μ*g·g^−1^; quercetin, 145 *μ*g·g^−1^; kaempferol, 236 *μ*g·g^−1^; quercetin-3-O-a-l-rhamnopyranosyl-(1-6)-b-d-glucopyranoside, 89 *μ*g·g^−1^; kaempferol-3-O-a-l-rhamnopyranoside, 101 *μ*g·g^−1^; and kaempferol-3-O-a-l-rhamnopyranoside-(1-6)-b-d-glucopyranoside, 99 *μ*g·g^−1^. The jacaric acid concentration (102 *μ*g·g^−1^) in the extract was determined from the curve of oleic acid.

### 3.2. Acute Toxicity

The acute oral toxicity test of ExJds in female rats did not show any toxic reaction or mortality and the extract was found to be safe up to a dose of 5000 mg·kg^−1^.

### 3.3. Body Weight

**Figure 1:**
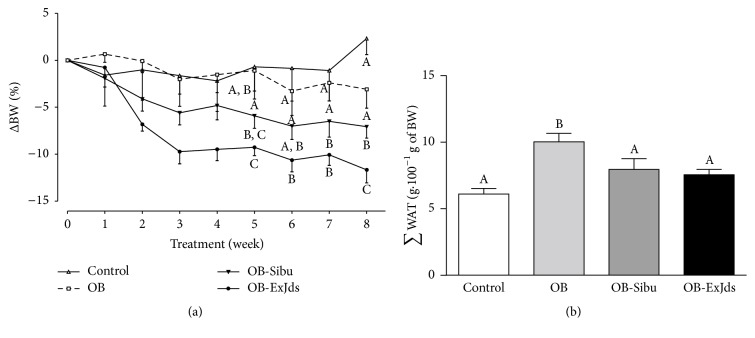
Body weight and white adipose tissue (WAT) measurement. (a) Change in body weight and (b) sum of WAT depots in Wistar fed with the control diet (control) and obese Wistar rats fed the hypercaloric diet and water (OB), sibutramine (2 mg·kg^−1^ body weight, OB-Sibu), or ExJds (400 mg·kg^−1^ body weight, OB-ExJds). Data are shown as mean ± standard error of the mean (SEM, *n* = 5) values. Different letters signify statistical difference at *P* < 0.05. ExJds, hydroethanolic extract of* Jacaranda decurrens *leaves.

Compared with the OB group, the obese Wistar rats showed a reduction in body weight of 12 or 7% (*P* < 0.001 and *P* < 0.05), respectively, at the end of the treatment. This result indicated that the effect of ExJds was superior to that of sibutramine (Figure 1(a)). The sum of the WAT depots in the OB group increased by 64% while ExJds and sibutramine reduced this parameter to levels that were similar to those found in the control diet-fed rats (Figure 1(b)).

### 3.4. Food Consumption and Water Intake Ingestion

**Figure 2:**
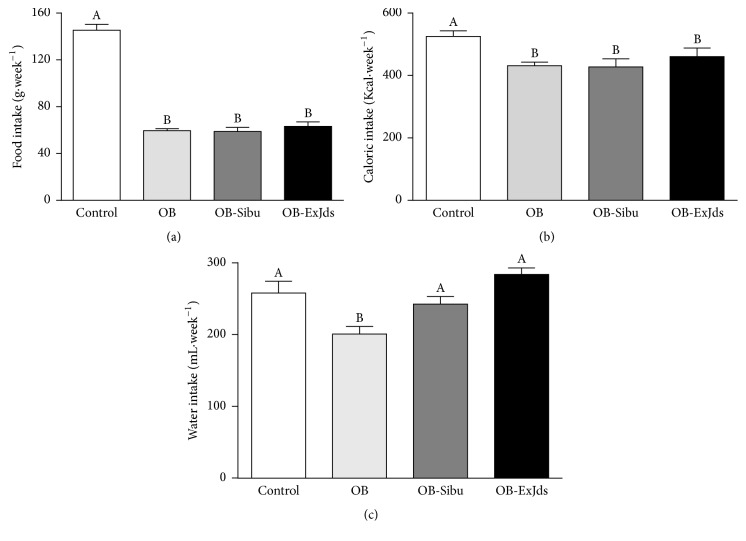
Food and water intake. Weekly ingestion of (a) chow and (b) calories and (c) water intake of Wistar rats fed control (control) and hypercaloric (OB) diets and treated with water, sibutramine (2 mg·kg^−1^ body weight, OB-Sibu), and ExJds (400 mg·kg^−1^ body weight, OB-ExJds). Data are shown as mean ± standard error of the mean (SEM, *n* = 5). Different letters signify statistical difference at *P* < 0.05. ExJds, hydroethanolic extract of* Jacaranda decurrens *leaves.

For the ExJds- and sibutramine-treated groups, no changes were observed in the weight of food (grams) or calories ingested (Figures 2(a) and 2(b)), although the levels were lower than those in the control group rats. However, the ExJds- and sibutramine-treated rats showed increased water intake (Figure 2(c)), restoring the values to those observed in the control group.

### 3.5. Fecal Excretion and Lipid Content (%)

**Figure 3:**
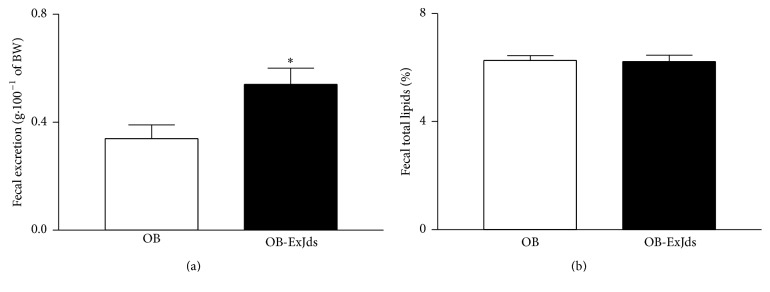
Analysis of feces weight and lipid content. (a) Feces excretion (gram of feces per 100 gram of body weight) and (b) percent lipid content (%) of obese Wistar rats fed the hypercaloric diet and treated with water (OB) and ExJds (400 mg·kg^−1^ body weight, OB- ExJds). Data are shown as mean ± standard error of the mean (SEM, *n* = 5); ^*∗*^ *P* < 0.05. ExJds, hydroethanolic extract of* Jacaranda decurrens *leaves.

The relative weight of the feces (gram of feces per 100 gram body weight) excreted by the rats treated with ExJds was greater than that of the feces of the control group rats (Figure 3(a)). Nevertheless, no differences were observed in the lipid percentage of the feces between the experimental groups (Figure 3(b)).

### 3.6. Tissue and Organ Weights and Hematological and Biochemical Parameters

The analysis of the different WAT depots showed that treatment with ExJds reduced the weight of the retroperitoneal depots, similar to treatment with sibutramine. In addition, ExJds reduced the subcutaneous WAT deposit (Table 1). No alterations were observed in the mass of muscle tissue, as shown in the soleus and extensor digitorum longus (EDL) muscles and organs evaluated, except a reduction in the liver mass of the Wistar rats treated with sibutramine (OB-Sibu) compared to that in the control animals. Both treatments reduced the serum total cholesterol and triglyceride levels compared to the levels recorded in OB animals (Table 2), which became similar to those of the control group animals. In addition, the animals with diet-induced obesity showed reduced serum hemoglobin levels. However, no differences were found in the hematological parameters between the obese groups.

## 4. Discussion

The prevalence of obesity and overweight has increased considerably worldwide, and the expenses incurred in their treatment and that of the associated diseases are expected to account for 9% of the total global health expenditure [13]. Therefore, the search for alternative treatments for obesity has increased, and the use of plants has become a viable treatment option for this disease.

Plants have diverse bioactive compounds that are responsible for their pharmacological actions. Jacaric, ursolic, and oleic acids, as well as luteolin, quercetin, and kaempferol, were identified and quantified in an extract of the leaves of* J. decurrens*. Of these compounds, only ursolic acid was found in the leaves of* J. decurrens* spp. [14] and in the roots of* J. decurrens *subsp.* symmetrifoliolata *[15]. The toxicity evaluation of elevated ExJds doses in the present study revealed no signs of toxicity or death among the treated animals. These results corroborate previous data indicating a lack of toxicity of the roots of* J. decurrens subsp. symmetrifoliolata *[15]. In addition, some compounds isolated from the same genus showed no toxicity when evaluated alone, such as jacaranone acid isolated from* Jacaranda copaia* [16].

Phenolic compounds present in various species of the genus* Jacaranda* [17] have been described in the literature as possible alternative treatments for obesity. Therefore, these compounds could have contributed to the reduction in fatty body mass observed in the animals treated with ExJds because they activate the noradrenergic pathway [18] and induce effects similar to those of sibutramine [19]. In addition, when present in food, phenolic compounds can reduce the proliferation and differentiation of preadipocytes, the accumulation of triglycerides, and can stimulate lipolysis and the oxidation of the adipocyte fatty acids [20]. These mechanisms could also have contributed to the antiobesity effects observed in the rats treated with ExJds.

Body mass reduction was accompanied by a decrease in the masses of the retroperitoneal and subcutaneous WAT deposits, as well as an increase in water intake. An increase in hydration favors metabolism and thereby contributes to accelerating the gastrointestinal transit. In addition, it can decrease the osmolarity of the gastrointestinal tract and exert a thermogenic effect mediated by an osmosensitive mechanism [21].

Although the effect observed could have been due to the synergism between the phenolic compounds present in the extract, the individual constituents have been described as antiobesity agents and modulators of the different parameters related to obesity. This has been observed with jacaric acid, which downregulated the expression of stearoyl-CoA desaturase, a key enzyme in fatty acid metabolism, in the liver of mice [22]. When administered alone, ursolic acid increased the activity of the lipolytic enzymes in primary cultures of adipocytes [23], inhibited the predifferentiation of the preadipocytes, stimulated lipolysis in the 3T3-L1 cells [24], and, in this study, reduced the total body mass and WAT* in vivo*. However, the administration of ursolic acid alone did not alter water intake [25], as was observed in the animals treated with ExJds, indicating the presence of other active compounds in the extract.

The dietary consumption of different amounts of oleic acid can result in dose-dependent beneficial effects on the regulation of lipid metabolism and contribute to body mass homeostasis [26]. Another constituent identified in the ExJds was luteolin, the derivatives of which have been reported to have antiadipogenic effects* in vitro* [27] and prevent diet-induced obesity in rats [28].

Several studies have shown the beneficial effects of quercetin [29–31], another flavonoid identified in the ExJds extract, which promotes alterations in the various parameters associated with obesity. Dong et al. [29] showed that the administration of a diet supplemented with quercetin reduced body mass in rats fed a fat-rich diet. In addition, quercetin acts as an antiadipogenic agent, inducing apoptosis in adipocytes [30]. Another plant species,* Aspalathus linearis*, contains quercetin as a major component and inhibits adipogenesis and the accumulation of triglycerides in adipocytes [31]. Studies carried out with kaempferol revealed that this flavonoid reduces adipogenesis in 3T3-L1 cells [32]. In addition, Bhattacharya et al. [33] demonstrated that flower extracts containing quercetin and kaempferol reduced the accumulation of fat in* Caenorhabditis elegans, *a model for obesity.

Another important parameter that could have contributed to the reduction in body mass of the animals treated with ExJds was the increase in fecal excretion. Since the extract promoted prebiotic effects, which have also been described for other plant-derived extracts [34], it likely had positive effects on the flora and thereby increased intestinal motility [35].

The reduction in adipose tissue was accompanied by a decrease in total cholesterol and serum triglyceride level, but no changes were observed in the skeletal muscle mass, which was evaluated at the end of the chronic treatment. In addition, none of the other organs investigated showed any signs of toxicity. Collectively, these data corroborate the absence of toxicity at elevated doses of ExJds because a reduction in muscle mass [36] and alterations in biochemical and hematological parameters [37] are markers of toxicity for numerous drugs.

## 5. Conclusions

In conclusion, these data show that the chemical constituents of the ExJds are nontoxic, and the body mass and WAT reduction observed were probably mediated by the increase in water intake and feces excretion in the rats treated with the ExJds.

## Figures and Tables

**Table 1:** White adipose tissue (WAT) depots of various groups of obese Wistar rats.

WAT	Control	OB		OB-Sibu		OB-ExJds	
	g·100 g^−1^	g·100 g^−1^	Δ (%)	g·100 g^−1^	Δ (%)	g·100	Δ (%)
Retroperitoneal	2.48 ± 0.23^a^	4.95 ± 0.35^b^	99	3.55 ± 0.52^a^	−28	3.46 ± 0.23^a^	−30
Epididymal	2.24 ± 0.13	2.92 ± 0.27	30	2.46 ± 0.17	16	2.24 ± 0.13	−23
Mesenteric	1.14 ± 0.11	1.90 ± 0.22	67	1.66 ± 0.20	−13	1.65 ± 0.27	−13
Subcutaneous	0.25 ± 0.02^a^	0.26 ± 0.01^a^	4	0.29 ± 0.02^a^	+11	0.20 ± 0.01^b^	−23

WAT depots (g·100 g^−1^ BW) of obese Wistar rats fed control diet (control), hypercaloric diet (OB), hypercaloric diet and sibutramine (2 mg·kg^−1^ BW, OB-Sibu), or hypercaloric diet and ExJds (400 mg·kg^−1^ BW, OB-ExJds). BW, body weight; WAT, white adipose tissue; Δ (%), variation in weight of WAT relative to that in the OB group. Data are shown as mean ± standard error of the mean (SEM, *n* = 5). Different letters signify statistical differences at *P* < 0.05.

**Table 2:** Tissue and organ mass and hematological parameters of nonobese (control) and obese (OB) Wistar rats.

Parameters	Control	OB	OB-Sibu	OB-ExJds
*Soleus* (g·100 g^−1^ BW)	0.030 ± 0.003	0.027 ± 0.002	0.027 ± 0.001	0.029 ± 0.003
EDL (g·100 g^−1^ BW)	0.011 ± 0.003	0.013 ± 0.001	0.013 ± 0.001	0.014 ± 0.001
Heart (g·100 g^−1^ BW)	0.204 ± 0.007	0.202 ± 0.008	0.191 ± 0.004	0.208 ± 0.010
Kidney (g·100 g^−1^ BW)	0.538 ± 0.016	0.475 ± 0.020	0.464 ± 0.023	0.480 ± 0.016
Liver (g·100 g^−1^ of BW)	2.617 ± 0.120^a^	2.74 ± 0.158^a^	2.26 ± 0.080^b^	2.74 ± 0.062^a^
Glycemia (mg·dL^−1^)	68.6 ± 6.1	70.0 ± 4.0	58.2 ± 2.6	66.0 ± 7.4
Total cholesterol (mg·dL^−1^)	46.2 ± 5.1^a^	75.6 ± 7.8^b^	45.4 ± 3.2^a^	48.2 ± 1.3^a^
Triglyceride (mg·dL^−1^)	101.8 ± 4.3^a^	222.4 ± 22.9^b^	82.6 ± 7.1^a^	99.6 ± 9.9^a^
Hematocrit (%)	52 ± 1	49 ± 1	48 ± 1	48 ± 1
Hemoglobin (g·dL^−1^)	15.1 ± 0.25^a^	13.9 ± 0.05^b^	13.8 ± 0.2^b^	13.5 ± 0.2^b^
Leukocyte (10^3^·*μ*L^−1^)	6.6 ± 0.8	7.3 ± 0.5	10.3 ± 2.0	7.9 ± 2.1

Nonobese (control) and obese (OB) Wistar rats were treated with water, sibutramine (2 mg·kg^−1^ BW, OB-Sibu), and ExJds (400 mg·kg^−1^ BW, OB-ExJds). BM, body mass; EDL, extensor digitorum longus. Data are shown as mean ± standard error of the mean (SEM, *n* = 5). Different letters indicate statistical significance at *P* < 0.05.
